# Effects of Nurses' Perceptions of Patient Safety Rules and Procedures on Their Patient Safety Performance: The Mediating Roles of Communication about Errors and Coworker Support

**DOI:** 10.1155/2023/2403986

**Published:** 2023-10-06

**Authors:** Ja-Kyung Seo, Seung Eun Lee

**Affiliations:** ^1^Department of Psychology, Graduate School, Yonsei University, Seoul, Republic of Korea; ^2^College of Nursing, Yonsei University, Seoul, Republic of Korea

## Abstract

**Aim:**

This study aimed to (a) examine the relationship between staff nurses' perceptions of patient safety rules and procedures and their patient safety performance and (b) investigate potential mediators of this relationship.

**Background:**

Implementation of effective management interventions to improve patient safety requires knowledge of the extent to which nurses' perceptions of a hospital's rules and procedures regarding patient safety affect their patient safety performance.

**Methods:**

This correlational study involved a secondary analysis of cross-sectional survey data collected from 1,053 staff nurses in South Korea. Structural equation modeling was employed to test the proposed mediation model. Five standardized measures were used to assess key study variables: patient safety compliance, patient safety participation, nurses' perceptions of patient safety rules and procedures, communication about errors, and coworker support. Cronbach's alpha values for the scales ranged from 0.82 to 0.90.

**Results:**

Nurses' perceptions regarding the usefulness and effectiveness of rules and procedures about patient safety were positively related to their patient safety performance, measured in terms of safety compliance and participation behaviors. Communication about errors and coworker support showed significant mediating effects on these relationships.

**Conclusions:**

The findings indicate that the implementation of effective and useful rules and procedures for improving patient safety would facilitate error communication and coworker support, enhancing nurses' patient safety performance. *Implications for Nursing Management*. Hospital administrators and nurse managers should consider how they can foster conditions in which nurses perceive rules and procedures regarding patient safety as useful and effective.

## 1. Introduction

The World Health Organization [1] has identified the development of clear safety guidelines and policies as a key action area for improving patient safety in healthcare organizations, as they establish rules and procedures to promote safety performance and reduce unsafe behaviors [2]. Research on organizational safety indicates that safety rules and procedures [3] have positive impacts on workers' safety performance and consequently result in better safety outcomes. Healthcare research has also suggested that nurses' positive perceptions of overall organizational policies facilitated their safety performance [4]. As nurses represent the largest healthcare workforce with the closest proximity to patients [5] and play an important role in improving patient safety across all healthcare settings [6], it is necessary to closely examine the association between safety-related rules and procedures and nurses' safety performance [7].

In the healthcare industry, a proactive metric of employees' safety performance is the assessment of safety compliance and participation [8, 9]. Safety compliance entails meeting the organization's mandatory safety requirements [10–12]. Examples of compliance behavior include following safety procedures and using proper personal protective equipment. Conversely, safety participation refers to employees' voluntary activities and efforts to support overall safety, such as attending voluntary safety meetings and raising safety concerns [12, 13].

Employees decide which behaviors are and are not expected based on the rules or policies that organizations implement [14]. According to signaling theory [15], such rules and policies signal employees as to whether their behaviors are consistent with organizational values [16]. Thus, it is reasonable to assume that nurses' perceptions of their organizations' rules and procedures pertaining to patient safety would positively correlate with their patient safety performance. Empirical studies [8, 10] have shown that positive beliefs and views about safety rules, procedures, and practices increase employees' safety performance by enhancing the safety climate. Therefore, we hypothesize that when hospital nurses perceive patient safety rules and procedures as both useful and effective, this perception directly leads to improved patient safety performance, manifested as heightened safety compliance and active safety participation.

Moreover, social psychology research suggests that workers, particularly those in occupations fraught with uncertainties and hazards such as healthcare, constantly seek social information from their colleagues to determine their work behaviors [17]. For nurses dealing with potential threats to patient safety on a daily basis, safety communication with coworkers and others is especially important. Safety communication is more than just exchanging information about workplace safety [18]; it is a two-way process of sending and receiving information that helps nurses to identify risks to patient safety and develop the attitudes and behaviors necessary to address them [19]. Our study evaluates this dimension of information exchange as communication about errors, which includes sharing information about previous errors, discussing ways to avoid them in the future, and being informed when organizational changes are made to improve patient safety [20]. Previous organizational research outside the healthcare context has highlighted the significant association between safety communication and safety performance [21], safety compliance [22], and safety participation [13]. Communicating about safety hazards, goals, and rules allows nurses to better understand the safety threats around them and their patients and develop appropriate behaviors to enhance patient safety [23].

Coworkers can serve as credible and appealing communicators about safety because of their operational experience and proximity [17]. Therefore, we predict that coworker support is another vital antecedent for nurses' patient safety performance. The social information processing (SIP) theory [24] suggests that sources of social information can be quantified according to their strength (power or importance), intimacy (proximity), and quantity (number of people involved) [25]. More specifically, this theory contends that the greater the importance, proximity, and number of people nearby, the more likely it is that individual behavior will be influenced by the social setting [26]. Based on the research findings discussed above, it is reasonable to assume that in the healthcare context, the salience, proximity, and quantity of coworkers who support patient safety impact nurses' safety compliance and safety participation. Consequently, we hypothesize that a positive correlation exists between increased coworker support and improved patient safety performance among nurses.

Given these considerations, the objectives of our study were twofold: (a) to investigate the effects of staff nurses' perceptions of patient safety rules and procedures on their patient safety performance, measured in terms of compliance and participation behaviors and (b) to examine whether communication about errors and coworker support play mediating roles in these relationships.

## 2. Methods

### 2.1. Setting and Sample

**Figure 1:**
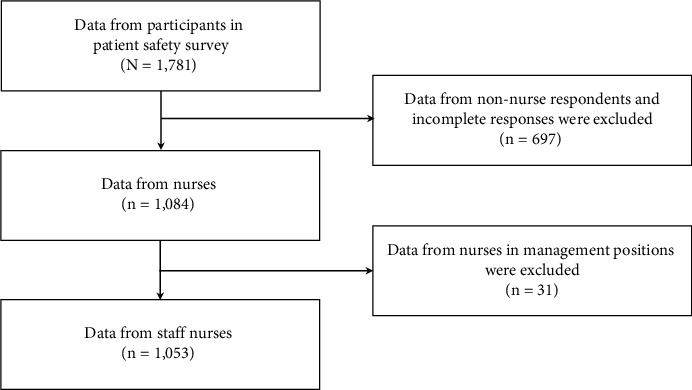
Flowchart for study sample.

This study employed a cross-sectional, correlational design. We conducted a secondary analysis of data from a patient safety culture survey conducted at a nonprofit acute care teaching hospital in South Korea. Every other year, the hospital conducts a survey of all its employees to assess their perceptions of its patient safety culture. This survey is guided by the Agency for Healthcare Research and Quality surveys for patient safety culture programs [27]. In October and November 2021, 2,200 hospital employees working in this hospital were invited to complete paper-and-pencil questionnaires. Because employees decided whether or not they would participate in the survey, a nonprobability sampling method was employed. The employees were assured of the confidentiality of survey participation and were informed about the voluntary nature of participation. The survey took approximately 15 minutes to complete. A total of 1,781 surveys were returned, representing a response rate of 81.0%. Among the respondents, 1,053 were staff nurses, and their responses were included in our data analysis. Data from non-nursing staff (e.g., physicians) and from nurses in management positions were excluded from the data analysis, as our study focused on staff nurse perceptions (see Figure 1). More detailed information about sampling is published in another article [9].

A sample size of 1,053 was considered adequate to achieve 0.8 power for detecting small effects among outcome, predictor, and mediator variables during structural equation modeling (SEM) analysis with bias-corrected bootstrapping [28]. The study design and procedures were consistent with the Declaration of Helsinki and received ethics approval from the University Health System Institutional Review Board (#4-2022-0339).

### 2.2. Measures

Data on the following demographic variables were collected: gender, age, employment status, work unit, hospital tenure, and unit tenure. The outcome, predictor, and mediator variables are described below; each was rated on a 5-point Likert scale ranging from 1 (*strongly disagree*) to 5 (*strongly agree*), with higher scores indicating higher levels of the construct.

#### 2.2.1. Outcome Variables

*Patient safety compliance* (three items) and *patient safety participation* (three items) were measured as outcome variables using two subscales of the Safety Behavior Scale [12] that were adapted to obtain a patient safety perspective. Items of the patient safety compliance subscale assessed the degree to which nurses followed essential, mandatory rules and procedures regarding patient safety, whereas the items of the patient safety participation subscale measured their level of voluntary participation in patient safety improvement. An example item for patient safety compliance is, “I use the correct patient safety-related procedures for carrying out my job.” An example item for patient safety participation is, “I voluntarily carry out tasks or activities that help to improve patient safety.” The higher the participant's scores, the greater the compliance with rules and procedures and voluntary participation in patient safety activities. The safety behavior scale showed construct validity [12], and in a previous Korean study [29], Cronbach's alpha values for the patient safety compliance and participation subscales were 0.94 and 0.94, respectively. In the present study, Cronbach's alpha values for patient safety compliance and participation were 0.86 and 0.87, respectively.

#### 2.2.2. Predictor Variable

The predictor variable, *nurses' perceptions of patient safety rules and procedures* (three items), was measured using a subscale of the safety climate scale [13] that was adapted to obtain a patient safety perspective. An example item is, “The patient safety procedures and practices in this organization are useful and effective.” Neal et al. [13] found that the subscale demonstrated good discriminant validity with respect to other subscales of the safety climate scale. Subsequently, Flatau Harrison et al. [30] further validated the instrument, reporting good validity and reliability. Cronbach's alpha values for the instrument were 0.84 in Flatau Harrison et al.'s [30] study and 0.90 in the present study.

#### 2.2.3. Mediator Variables

One mediator variable, *communication about errors* (three items), was assessed using the communication about errors subscale of the Korean version of the Hospital Survey on Patient Safety Culture 2.0, which demonstrated construct validity and acceptable reliability in Korean nurse populations [31]. An example item of this subscale is, “When errors happen in this unit, we discuss ways to prevent them from happening again.” Cronbach's alpha values for this subscale were 0.83 in Lee and Dahinten's [31] study and 0.87 in the present study. A second mediator variable, *coworker support* (three items), was assessed using a scale developed by Tucker and colleagues that showed good reliability and validity [25]. An example item of this scale is, “My colleagues encourage each other to work safely.” Cronbach's alpha values for this scale were 0.90 in Tucker et al.'s [25] study and 0.82 in the present study.

### 2.3. Data Analysis

We first analyzed descriptive statistics for the demographic characteristics of the participants and the study variables. Second, Pearson's bivariate correlations were calculated to assess associations between the study variables. Next, we checked for the variance inflation factor (VIF), skewness, and kurtosis to determine whether multicollinearity and normality were violated. The statistical analyses were conducted using SPSS IBM 25.0, and the level of statistical significance was set at 0.05.

Mplus version 8.6 was used to conduct SEM analysis, with nurses' patient safety compliance and participation as the dependent variables, nurses' perceptions of rules and procedures about patient safety as the independent variable, and communication about errors and coworker support as the mediators of the relationships. The robust maximum likelihood estimator was used to estimate the model, and the following indices of model fit were chosen to evaluate the measurement and structural models: the standardized root mean square residual (SRMR <0.08), comparative fit index (CFI >0.90), Tucker–Lewis index (TLI >0.90), and root mean square error of approximation (RMSEA <0.08) [32]. Work unit was chosen as a control variable because previous research has shown significant differences by work unit in nurses' safety-related perceptions and behaviors [33]. We also controlled for hospital tenure due to its positive correlation with patient safety participation (*r* = 0.08, *p* < 0.05). Finally, we tested the significance of the direct and indirect effects of nurses' perceptions of patient safety rules and procedures on the two forms of patient safety performance through communication about errors and coworker support. To do so, we implemented bootstrapping with 10,000 bootstrap samples and 95% bias-corrected confidence intervals (CIs), as suggested by Preacher and Hayes [34].

## 3. Results

### 3.1. Sample Characteristics

Most study participants were female (94.6%) and in the 20- to 29-year age group (58%; Table 1). Approximately 95% of participants held permanent, full-time positions. The average unit tenure was 3.9 years, and the mean hospital tenure was 7.1 years. The participants most commonly worked in medical, surgical, or medical-surgical units, followed by critical care, perioperative care, and other units.

### 3.2. Correlations between Key Study Variables

All key study variables were significantly and positively correlated with each other. Nurses' perceptions of rules and procedures regarding patient safety showed correlations with patient safety compliance (*r* = 0.52, *p* < 0.001) and patient safety participation (*r* = 0.54, *p* < 0.001). Communication about errors was correlated with patient safety compliance (*r* = 0.44, *p* < 0.001) and participation (*r* = 0.37, *p* < 0.001), and coworker support showed correlations with patient safety compliance (*r* = 0.56, *p* < 0.001) and participation (*r* = 0.45, *p* < 0.001) (see Table 2).

### 3.3. Hypothesis Testing: Mediating Effects of Communication about Errors and Coworker Support

**Figure 2:**
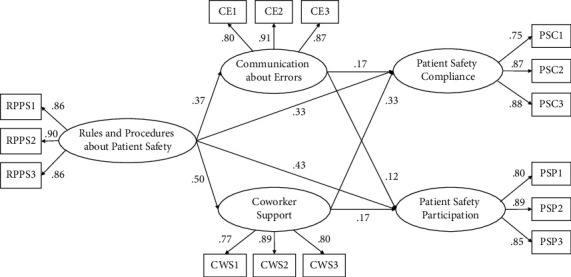
Final model: standardized model paths and standardized item loadings. *Note*. Hospital tenure and dummy variables were controlled for in the model. All paths and loadings were significant at the 0.05 level. RPPS, (nurses' perceptions of) rules and procedures regarding patient safety; CE, communication about errors; CWPS, coworker support; PSC, patient safety compliance; PSP, patient safety participation.

The VIFs between the predictor and mediators ranged from 1.31 to 1.44, indicating no potential multicollinearity problems [35]. The skewness or kurtosis levels of the five study variables ranged from −0.42 to 0.84 and did not exceed the threshold of an absolute value of 2 [36], indicating a nonsignificant deviation from normality. Next, a confirmatory factor analysis (CFA) was performed to test the fit of the five-factor measurement model and assess the degree to which the observed items loaded onto their respective latent variables. The measurement model showed a good fit to the data: *χ*^2^ (80) = 285.964, CFI = 0.981, TLI = 0.975, RMSEA = 0.049, and SRMR = 0.032. Standardized loadings relating indicators to their respective latent factors were all statistically significant (*p* < 0.001) and ranged from 0.748 to 0.908 (see Figure 2).

Our hypothesized partial mediation model showed a good fit to the data: *χ*^2^ (210) = 522.151, CFI = 0.972, TLI = 0.965, RMSEA = 0.038, and SRMR = 0.037. To determine the best-fitting model, the direct paths from nurses' perceptions of patient safety rules and procedures to both types of patient safety performance were removed to specify a full mediation model. This model also provided a good fit to the data: *χ*^2^ (212) = 669.392, CFI = 0.958, TLI = 0.950, RMSEA = 0.045, and SRMR = 0.058. A chi-square difference test was performed to compare the full mediation model to our original partial mediation model. The test results demonstrated that the partial mediation model was a significantly better fit than the full mediation model (∆*χ*^2^ = 147.241, ∆*df* = 2, *p* < 0.001) (see Figure 2).

As displayed in Table 3, the results of the SEM analysis showed that nurses' perceptions of patient safety rules and procedures significantly affected the mediating roles of communication about errors (*β* = 0.369, *p* < 0.001) and coworker support (*β* = 0.501, *p* < 0.001). In addition, nurses' perceptions of patient safety rules and procedures had significant and positive direct effects on patient safety compliance (*β* = 0.325, 95% CI = 0.202–0.401) and participation (*β* = 0.429, 95% CI = 0.326–0.543), even after accounting for mediating effects. Both types of patient safety performance were significantly influenced by both mediators. Specifically, patient safety compliance was significantly affected by communication about errors (*β* = 0.169, *p* = 0.001) and coworker support (*β* = 0.332, *p* < 0.001), and patient safety participation was also significantly affected by communication about errors (*β* = 0.124, *p* = 0.014) and coworker support (*β* = 0.174, *p* = 0.006).

Next, a 95% CI of the parameter estimates was calculated using 10,000 samples from the raw data. The indirect effects of nurses' perceptions of patient safety rules and procedures on patient safety compliance through communication about errors (*β* = 0.062, 95% CI = 0.025–0.092) and coworker support (*β* = 0.166, 95% CI = 0.101–0.210) were significant. Furthermore, the indirect effects of nurses' perceptions of patient safety rules and procedures on patient safety participation through communication about errors (*β* = 0.046, 95% CI = 0.010–0.081) and coworker support (*β* = 0.087, 95% CI = 0.031–0.145) were significant.

## 4. Discussion

To the best of our knowledge, this is the first nursing study to examine the direct and indirect effects of nurses' perceptions of rules and procedures regarding patient safety on their performance with respect to patient safety. We found that these perceptions directly affected the outcome variable with a larger effect size than that of the indirect paths. This suggests that implementing rules and procedures supporting patient safety in healthcare organizations can significantly improve nurses' patient safety performance. The scale used to assess nurses' perceptions of patient safety rules and procedures in this study emphasizes their levels of usefulness and effectiveness from nurses' perspective. In a recent study, Vu et al. [37] found that perceived usefulness with respect to safety rules and procedures can improve individuals' safety performance, and our results also show that when nurses perceived their organization's rules and procedures to be useful and effective, they exhibited both mandatory compliance and voluntary participation for patient safety. This finding is noteworthy, as in the safety literature, the effect of safety rules and procedures has typically been evaluated as a subfactor of larger constructs such as safety climate [38] and workplace safety management practices [39]. In fact, rules and procedures can be used as signals or cues to inform employees about behaviors expected in organizations [14], and our findings revealed that useful and effective rules and procedures alone could serve as valuable signals to generate desired employee performance in terms of patient safety.

Our mediation analyses showed that positive perceptions of patient safety rules and procedures encouraged nurses to communicate about errors, which in turn increased their patient safety performance. The significant mediating effect of communication about errors indicates that once rules and procedures supporting patient safety are successfully implemented in a hospital, nurses become more willing to engage in safety communication. A clear set of rules and procedures functions as shared knowledge among staff, and this pooled knowledge can initiate communication about patient safety-related information, ideas, and concerns. This finding is consistent with research results from industrial organizational psychology and sports psychology demonstrating that shared knowledge among team members lays the foundation for active team communication [40]. Our findings also support earlier study results stressing the significance of knowledge sharing for open communication [41, 42] and for medical staff's safety behaviors [43]. Our findings further highlight the importance of explicit communication and feedback about errors in healthcare by identifying the positive association between safety communication and safety performance.

In addition, our mediation analyses provided evidence of the importance of coworker support for nurses' patient safety performance. The findings indicated that when nurses recognized the usefulness and effectiveness of patient safety rules and procedures in their organizations and followed them, other nurses observed their behavior and did the same. This result is consistent with research conducted outside the healthcare industry reporting that a “horizontal social contagion effect” from coworkers affected employees' safety performance [17]. In our study, the association between coworker support and nurses' patient safety compliance was stronger than that between coworker support and their patient safety participation. Moreover, the indirect effect of nurses' perceptions of rules and procedures regarding patient safety on safety compliance through coworker support was the strongest among the four indirect paths examined. These results support the relevance of the SIP theory [23] in the healthcare context, as the theory posits that information gained from intimate coworkers has a profound effect on instilling descriptive norms regarding patient safety. Previous research [44] has already identified a positive association between coworker support and nurses' safety performance, and our study provides additional concrete evidence of this relationship. Because nurses spend a large amount of time in uncertain and risky healthcare environments, the assurance and support they receive from coworkers could contribute to their confidence to perform safety behaviors.

Finally, our study broadens patient safety research by examining the effects of newly identified antecedents on nurses' patient safety performance. Although safety research has been widely conducted across industries, the scope of the antecedents examined has mostly been limited to organizational factors such as leadership and job design [45] and individual factors such as knowledge and motivation [46]. Regarding the patient safety context, prior studies have thus far discovered that management commitment to safety, safety communication and feedback, and patient safety climate are positively related to patient safety behavior [47, 48]. Our study newly identifies nurses' perceptions of patient safety rules and procedures as predictors, and error communication and coworker support as mediators for nurses' patient safety performance. In doing so, it expands the safety performance literature by offering new ways to improve nurses' patient safety performance in healthcare organizations. In particular, our findings could provide a foundation for practical intervention, as healthcare organizations can and should implement rules and procedures that nurses recognize as improving patient safety.

### 4.1. Limitations

This study had limitations that should be noted. First, because our study had a cross-sectional design, causal conclusions could not be drawn. Future researchers should design longitudinal studies to determine causal relationships between the study variables and to further explore whether nurses' perceptions of patient safety rules and procedures, communication about errors, and coworker support contribute to their long-term patient safety performance. Second, our data were obtained from a single hospital in South Korea, which may limit the generalizability of our findings to other populations and settings. Finally, our data were collected through self-reporting, which may have resulted in response bias. Although the CFA results indicated good data fit for the measurement model, response bias may still have been present. Despite these limitations, this study adds new evidence to a sparse area of the literature by identifying the effects of nurses' perceptions of rules and procedures regarding patient safety on their patient safety performance as well as mediators of these effects.

## 5. Conclusions

Complying with safety rules and taking both mandatory and voluntary actions to address patient safety risks are crucial, emphasizing the need to identify strategies for enhancing nurses' patient safety performance. Our mediation model demonstrates that when nurses have positive perceptions of their hospital's rules and procedures regarding patient safety, their patient safety performance is improved, and this improvement is facilitated by communication about errors and coworker support. Moreover, our findings suggest that effective hospital implementation of well-reasoned safety measures could lead nurses to view these initiatives positively, resulting in heightened safety compliance and participation; this process could be further catalyzed by open dialogue about mistakes and robust coworker backing. Thus, hospital administrators and nurse managers should recognize and take advantage of the potentially beneficial role of rules and procedures in nurses' patient safety performance.

## 6. Implications for Nursing Management

Nurses' patient safety performance may be driven by the perceived usefulness and effectiveness of patient safety-related rules and procedures. Therefore, hospital management should take steps to strengthen nurses' perceptions of the usefulness and effectiveness of these guidelines. For instance, nurse managers can help their staff become familiar with safety rules and procedures by providing detailed guidance on how they should be followed. Also, to emphasize the value of the rules and procedures, hospital management should clearly explain why the safety measures need to be followed, provide easy access to equipment and tools needed for proper execution of procedures, and proactively offer resources to alleviate any potential difficulties in carrying out procedures.

As for coworkers, nurses should encourage each other to work safely and provide feedback to each other on their safety performance [25]. To make this possible, nurse staff and managers alike should make an effort to create a psychologically safe work environment by being open to feedback, sharing failures through transparent communication, and appreciating new perspectives and ideas [49]. In addition, nurse managers should hold regular staff safety meetings to share patient safety experiences and create a sense of mutual trust and support among nurses. Furthermore, nurse managers should facilitate information-sharing platforms and support networks among nurses so that they can freely discuss patient safety problems with coworkers face-to-face [50]. All these actions will serve to promote hospital nurses' acceptance of and adherence to rules and procedures regarding patient safety, their communication about errors and mutual support, and improved patient safety performance.

## Figures and Tables

**Table 1:** Sample demographic characteristics (*N* = 1,053).

Variables	Category	*n*	%	*M*	SD
Gender (*n* = 1,046)	Male	56	5.4		
	Female	990	94.6		
Age (*n* = 1,046)	20–29	606	57.9		
	30–39	254	24.3		
	40–49	146	14.0		
	≥50	40	3.8		
Employment status (*n* = 1,012)	Permanent	964	95.3		
	Temporary	48	4.7		
Work unit (*n* = 1,033)	Medical, surgical, or medical-surgical	454	44.0		
	Critical care	188	18.2		
	Perioperative	65	6.3		
	Others^a^	346	33.5		
Hospital tenure in years				7.1	7.4
Unit tenure in years				3.9	4.4

*Note*. *M*, mean; SD, standard deviation. Sample size varied because of missing data. ^a^Others included emergency department, rehabilitation, pediatrics, obstetrics, psychiatry, outpatient, urology, labor and delivery, and multiple units.

**Table 2:** Correlations and descriptive statistics for key study variables (*N* = 1,053).

Variables	1	2	3	4	5
(1) Patient safety rules and procedures	—				
(2) Communication about errors	0.40^*∗∗*^	—			
(3) Coworker support	0.44^*∗∗*^	0.49^*∗∗*^	—		
(4) Patient safety compliance	0.52^*∗∗*^	0.44^*∗∗*^	0.56^*∗∗*^	—	
(5) Patient safety participation	0.54^*∗∗*^	0.37^*∗∗*^	0.45^*∗∗*^	0.72^*∗∗*^	—

*M*	3.62	4.02	3.92	3.88	3.74
SD	0.62	0.61	0.57	0.57	0.63

*Note*. 1, nurses' perceptions of patient safety rules and procedures; *M*, mean; SD, standard deviation. ^*∗∗*^*p* < 0.01.

**Table 3:** Standardized direct and indirect effects for the hypothetical model (*N* = 1,053).

Path	*b*	*p*	95% CI (lower, upper)
Direct effects			
Rules and procedures regarding patient safety ⟶ patient safety compliance	0.325	<0.001	[0.202, 0.401]
Rules and procedures regarding patient safety ⟶ patient safety participation	0.429	<0.001	[0.326, 0.543]
Indirect effects			
Rules and procedures regarding patient safety ⟶ communication about errors ⟶ patient safety compliance	0.062	0.001	[0.025, 0.092]
Rules and procedures regarding patient safety ⟶ communication about errors ⟶ patient safety participation	0.046	0.012	[0.010, 0.081]
Rules and procedures regarding patient safety ⟶ coworker support ⟶ patient safety compliance	0.166	<0.001	[0.101, 0.210]
Rules and procedures regarding patient safety ⟶ coworker support ⟶ patient safety participation	0.087	0.001	[0.031, 0.145]

*Note*. Rules and procedures regarding patient safety refer to nurses' perceptions of patient safety rules and procedures; CI, bias-corrected confidence interval.

## Data Availability

The data used to support the findings of this study have not been made available because of confidentiality.
